# β-Catenin Is Required for the Tumorigenic Behavior of Triple-Negative Breast Cancer Cells

**DOI:** 10.1371/journal.pone.0117097

**Published:** 2015-02-06

**Authors:** Jinhua Xu, Jenifer R. Prosperi, Noura Choudhury, Olufunmilayo I. Olopade, Kathleen H. Goss

**Affiliations:** 1 Department of Surgery and Comprehensive Cancer Center, University of Chicago, Chicago, IL 60637, United States of America; 2 Department of Medicine, University of Chicago, Chicago, IL 60637, United States of America; 3 School of Medicine, Jianghan University, Wuhan, Hubei 430056, P.R. China; 4 Department of Biochemistry and Molecular Biology, Indiana University School of Medicine—South Bend, South Bend, IN 46530, United States of America; Van Andel Institute, UNITED STATES

## Abstract

Our previous data illustrated that activation of the canonical Wnt signaling pathway was enriched in triple-negative breast cancer and associated with reduced overall survival in all patients. To determine whether Wnt signaling may be a promising therapeutic target for triple-negative breast cancer, we investigated whether β-catenin was necessary for tumorigenic behaviors *in vivo* and *in vitro*. β-catenin expression level was significantly reduced in two human triple-negative breast cancer cell lines, MDA-MB-231 and HCC38, using lentiviral delivery of β-catenin-specific small hairpin RNAs (shRNAs). Upon implantation of the cells in the mammary fat pad of immunocompromised mice, we found that β-catenin shRNA HCC38 cells formed markedly smaller tumors than control cells and grew much more slowly. In *in vitro* assays, β-catenin silencing significantly reduced the percentage of Aldefluor-positive cells, a read-out of the stem-like cell population, as well as the expression of stem cell-related target genes including *Bmi-1* and *c-Myc*. β-catenin-knockdown cells were also significantly impaired in their ability to migrate in wound-filling assays and form anchorage-independent colonies in soft agar. β-catenin-knockdown cells were more sensitive to chemotherapeutic agents doxorubicin and cisplatin. Collectively, these data suggest that β-catenin is required for triple-negative breast cancer development by controlling numerous tumor-associated properties, such as migration, stemness, anchorage-independent growth and chemosensitivity.

## Introduction

Triple-negative breast cancer (TNBC), including basal-like breast tumors, is an aggressive tumor subtype that is distinguished from other breast cancer subtypes by its gene expression profiles, histopathological features, and clinical characteristics [1–3]. These tumors can be particularly challenging clinically because, although these tumors respond well to chemotherapy at least initially, they often develop drug resistance [4]. By virtue of TNBC lacking estrogen receptor (ER), progesterone receptor (PR) and HER2 expression, there are no targeted biological therapies currently used as standard treatment for these cancers [1, 5]. Therefore, an important goal of research in the TNBC field is to identify those signaling pathways that drive tumor behavior and may also serve as potential therapeutic targets.

The canonical Wnt signal transduction pathway is activated in several tumor types, including colorectal and breast cancer [6–8]. Among other mechanisms, the major effector of the canonical Wnt pathway, β-catenin, is stabilized in tumors primarily via Wnt ligand overexpression, down-regulation of Wnt ligand antagonists, or loss of the APC tumor suppressor [9]. As a consequence of its stabilization, β-catenin translocates to the nucleus where it controls gene expression through its association with members of the T cell factor (TCF) family of transcription factors. Some of the β-catenin/TCF transcriptional targets implicated in tumor initiation and progression include the cell cycle regulators *cyclin D1* [10] and *c-Myc* [11], stem cell gene *Bmi-1* [12], matrix metalloproteinase *Mmp-7* [13], and Wnt pathway component *Axin2* [14]. Our laboratory and others have demonstrated that the Wnt pathway is more frequently activated in TNBC than in other breast cancer subtypes (e.g., ER-positive cancers) [15–17]. Moreover, nuclear and cytosolic accumulation of β-catenin, but not membrane-associated β-catenin, is associated with reduced overall survival in all breast cancer patients [16]. We sought to determine the impact of Wnt/β-catenin pathway activation on the malignant and metastatic potential of TNBC cells *in vitro and in vivo.* In the present study, our group analyzed the role of β-catenin in the tumorigenic properties of TNBC cell lines to address its potential utility as a therapeutic target in this tumor type.

## Materials and Methods

### Ethics Statement

All procedures were performed with prior approval (protocol #71828) from the University of Chicago Institutional Animal Care and Use Committee, which has full accreditation from the Association for Assessment and Accreditation of Laboratory Animal Care.

### Cell lines and β-catenin knockdown

MDA-MB-231 and HCC38 cell lines were from ATCC and cultured in RPMI 1640 media (Invitrogen, Grand Island, NY) supplemented with 10% FBS and 1% penicillin/streptomycin. The stable knock down lines of MDA-MB-231 and HCC38 were established by infection of MISSION shRNA Lentiviral Transduction Particles against CTNNB1 (Sigma-Aldrich, TRCN0000003845 and TRCN0000003846) and selected with puromycin (1ug/ml).

### Western blotting

Total cell lysates were extracted with 50 mM Tris pH7.5, 0.1% IGEPAL, 100 mM NaCl, 1 mM MgCl_2_, 5 mM EDTA and protease inhibitors and sonicated. 25 µg of lysate from each cell line was separated on a 10% SDS-PAGE gel and transferred to Immobilon P membrane (Millipore). Blots were probed with 1:1000 anti-β-catenin monoclonal antibody (BD Biosciences), 1:1000 anti-active (unphosphorylated) β-catenin (EMD Millipore) and 1:1000 anti-actin monoclonal antibody (Sigma) in blocking buffer (5% nonfat dried milk in TBST).

### TOPFlash reporter assays

Cells were plated in a 24-well plate at a density of 1x10^4^ cells/well and transfected using Optifect (Invitrogen) with 2.6 μg of *pTOPflash* or *pFOPflash* (obtained from H. Clevers at the Hubrecht Insitute) along with *pRL-TK* (Promega) for determination of transfection efficiency. Lysates were harvested after 48 hrs and analyzed using the Dual Luciferase Assay System kit (Promega). Luciferase activity was normalized for transfection efficiency and graphed as ratio of TOPflash/FOPflash activity. Mutant β-catenin^S37A^ was from Stephen Byers at Georgetown University. The breast cancer lines were transfected with stabilized mutant β-catenin^S37A^ as described previously [18]. Expression was verified by GFP fluorescence and Western blotting with a HA antibody (Santa Cruz).

### Cell growth, migration and soft agar growth assays

Cell growth was analyzed with MTS assay using CellTiter 96 AQueous One Solution Cell Proliferation Assay (Promega, WI) following the protocol. Cells were seeded in 96-well culture plates at a density of 5×10^3^ cells/well. Cell viability was assessed by adding 3-(4,5-dimethylthiazol-2-yl)-5-(3-carboxymethoxyphenyl)-2-(4-sulfophenyl)-2H-tetrazolium (MTS) to the culture medium and incubated for 4 h at 37 °C. The optical density was measured at 490 nm using Synergy 2 plate reader (Biotek, VT). Cell migration was measured by wound healing assays as described [18]. Briefly, cells were plated at a density of 1 x 10^5^ cells per well in a 6-well plate were grown to confluence, at which time they were scratched with a micropipette tip. Phase-contrast images were captured with a Zeiss Axiovert 35 inverted microscope using AxioVision REL 4.6 imaging software (Zeiss) every 12 hr. The area of three representative wounds for each cell line was quantified at each time point using Image J software (NIH). Anchorage-independent growth soft agar assays were performed by seeding 4 x 10^3^ cells in 0.3% agarose as described [19]. The colonies were quantified at day 14 from five fields in triplicate wells.

### Stem cell assays

ALDEFLOUR assays (Stemcell) were used. 5x10^5^ cells were stained with ALDEFLOUR reagent or ALDEFLOUR reagent plus ALDEFLOUR inhibitor. Stained cells were sorted by the University of Chicago Flow Cytometry Core Facility.

### Quantitative real-time PCR

Gene expression changes were analyzed by real-time RT-PCR as described [20] except ribosomal RNA 18S was used as a normalization control. The primer sequences for *c-Myc*: Forward 5’-CAG CTG CTT AGA CGC TGG ATT T-3’; Reverse 5’-ACC GAG TCG TAG TCG AGG TCA T-3’.The primer sequences for *Axin2*: Forward 5’-CTG GCT TTG GTG AAC TGT TG-3’; Reverse 5’- AGT TGC TCA CAG CCA AGA CA-3’. The primer sequences for *Bmi-1*: Forward 5’-CTC CCA ACT GGT TCG ACC TT-3’; Reverse 5’-CGG TTT CCA TAT TTC TCA GT-3’. The primer sequences for ribosomal RNA 18S has been reported [18]. The fold change in target gene relative to the control was determined by 2^-∆∆Ct^ method.

### Xenograft transplantation assays

5 x 10^6^ parental, scrambled control shRNA or β-catenin-knockdown cells were injected with Matrigel (BD Biosciences) into the mammary fat pad of Nu/Nu female mice (n = 10 per group). Tumors were measured biweekly with calipers after implantation, and the animals were sacrificed 11 weeks later. Tumor volume was calculated as described previously [8]. After euthanasia, tumors were fixed, sectioned and stained with hematoxylin and eosin (H&E); images were captured on a Leica DMLB microscope using Qcapture Pro 6.0 imaging software.

### Therapeutic response of TNBC cells

β-catenin-knockdown and control HCC38 cells were treated with 0.5 μM doxorubicin or 3.0 μM cisplatin for 24 hrs and subjected to an Caspase 3/7 Glo assay (Promega, WI, USA). Relative sensitivity was shown as β-catenin-knockdown over control.

### Statistical analysis

All experiments were performed at least in triplicate, and statistical significance was defined as a p value of <0.05 using a one-way ANOVA with a Student’s t-test post-test, unless noted otherwise. For quantification, means are shown with standard deviation (SD).

### Results

**Figure 1:**
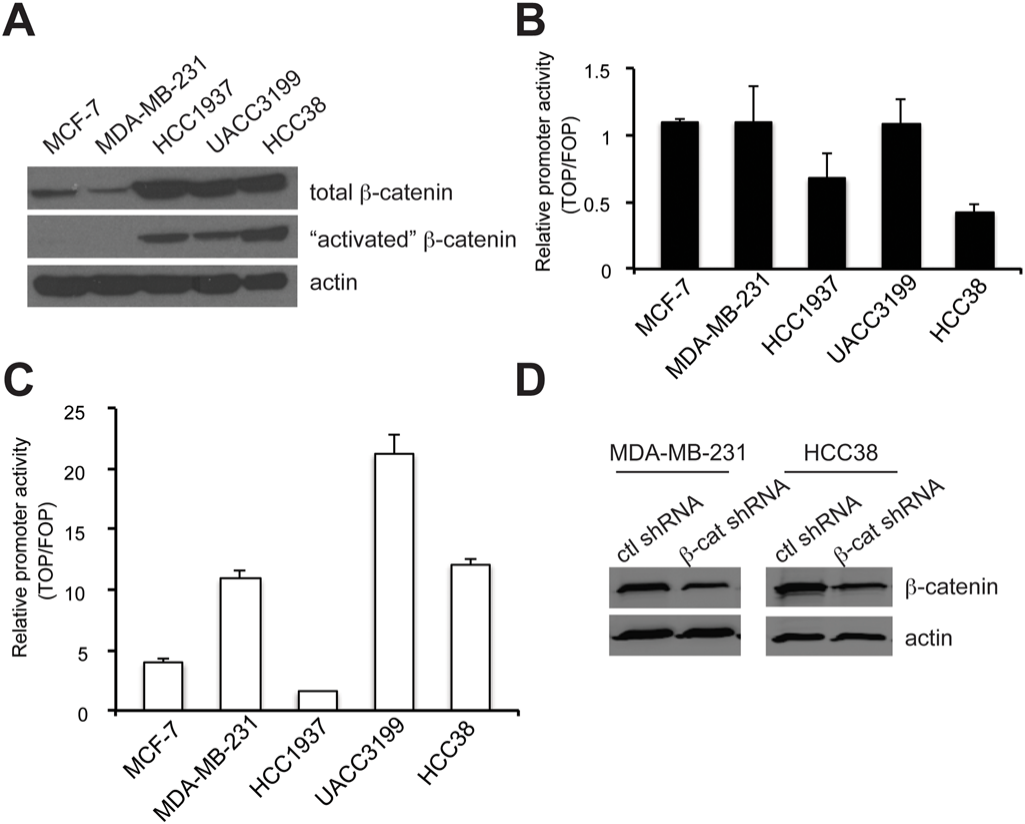
β-catenin is overexpressed in TNBC cell lines. (A) Western blots of total lysates were probed with antibodies against total β-catenin and active β-catenin (EMD Millipore). (B) Basal TCF reporter activity was measured upon transfection with pTOPFlash or pFOPFlash for 48 hr. Normalized luminescence values are presented as TOP/FOP ratio. (C) TCF reporter activity of the cells was measured upon transient transfection of mutant β-cateninS37A. Values were normalized to vector-transfected cells. (D) β-catenin expression was stably knocked down in MDA-MB-231 and HCC38 cells by lentiviral transduction of *CTNNB1*-specific or scrambled control shRNAs, and Western blotting was performed as in A.

In order to identify *in vitro* models of TNBC that recapitulate some of the features of human tumors with respect to Wnt signaling, several breast cancer cell lines were analyzed for total and “activated”, or non-phosphorylated, β-catenin by Western blotting. Total and “activated” β-catenin were expressed at high levels in HCC1937, UACC3199 and HCC38 TNBC cell lines compared to the ER-positive MCF-7 and another TNBC line MDA-MB-231 (Fig. 1A). Interestingly, despite the high levels of “activated” β-catenin, none of the cell lines demonstrated significant basal TCF reporter activity (Fig. 1B). However, reporter activity was induced in most of the cell lines upon transient overexpression of a stabilized S37A mutant of β-catenin (Fig. 1C). There was significant variability among the cell lines in the extent of pathway activation in response to stabilized β-catenin overexpression, suggesting that there are other factors, beyond the levels of exogenous or endogenous β-catenin, that contribute to β-catenin/TCF transcriptional activity. Two TNBC cell lines with similar levels of induced TCF reporter activity, MDA-MB-231 and HCC38, were selected to test the function of β-catenin using RNA interference. β-catenin expression was stably knocked down in each cell line by lentiviral transduction of β-catenin-specific shRNA and selection with puromycin. Scrambled shRNA was used as control. Western blotting with anti-β-catenin antibodies illustrated that there was a significant reduction in β-catenin levels in the stable β-catenin shRNA cell lines compared to controls (Fig. 1D).

**Figure 2:**
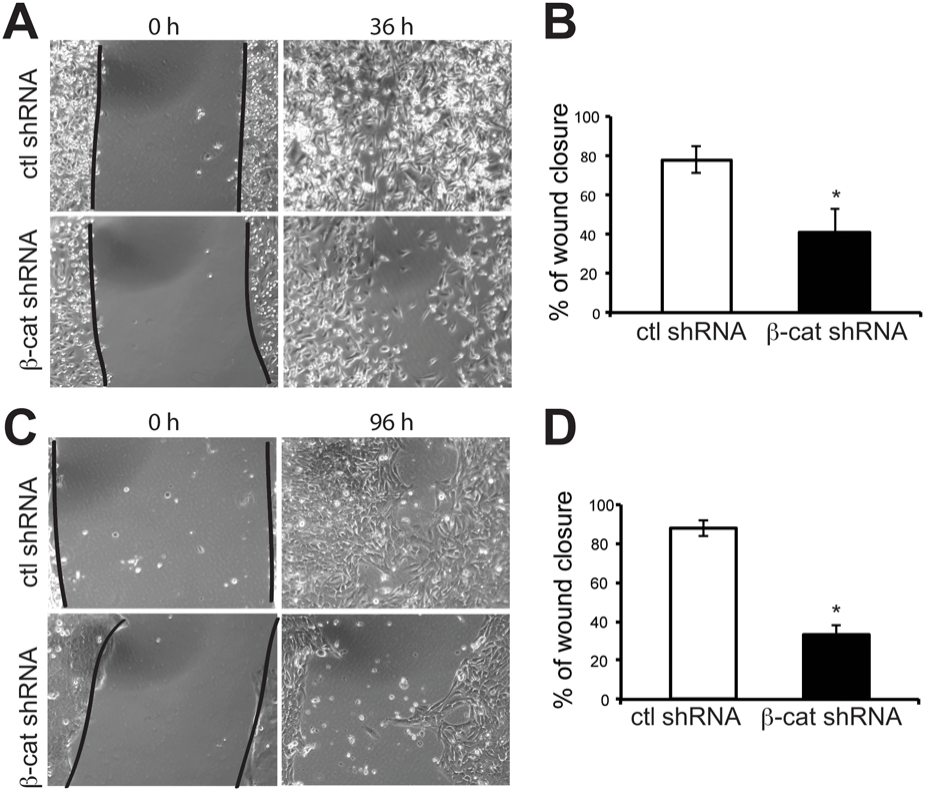
Stable knockdown of β-catenin suppresses migration of triple-negative breast cancer cells. Confluent monolayers of β-catenin-knockdown or control MDA-MB-231 (A-B) and HCC38 (C-D) cells were scratched with a micropipette tip and imaged up to 5 days after on an inverted microscope every 12 hr. Quantification was performed on two separate experiments performed in triplicate. Wound area was quantified with Image J software. *p<0.01.

**Figure 3:**
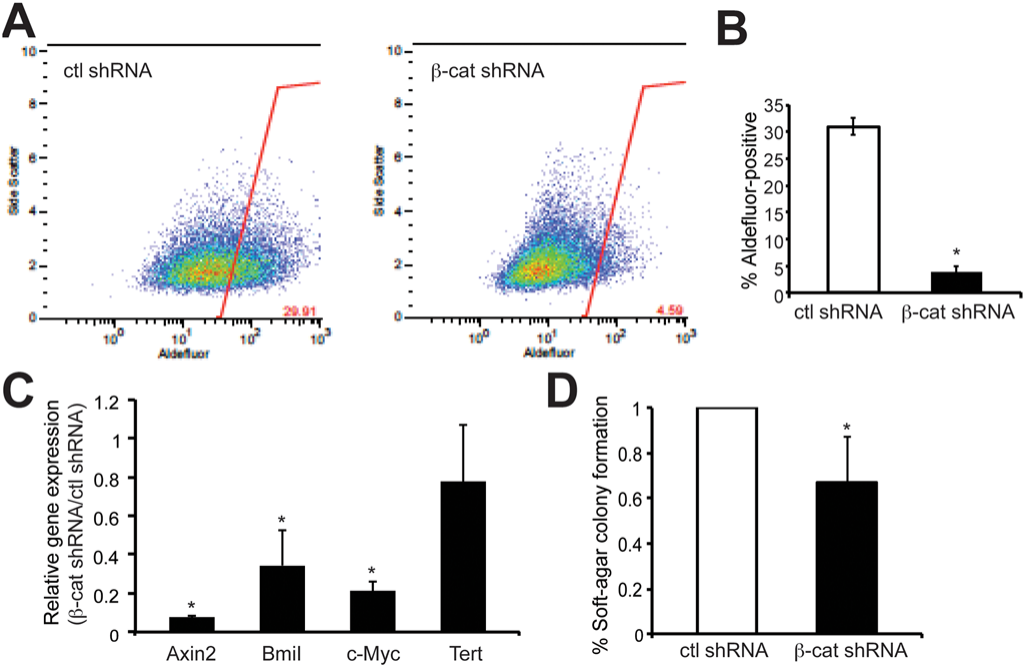
β-catenin is required for stemness and the colony formation capacity of HCC38 TNBC cells. (A-B) The stem-like population of β-catenin-knockdown and control HCC38 cells was measured using the ALDEFLOUR assay (Stemcell) and flow cytometry. Data show means ± SD of two independent experiments. *p<0.01. (C) Gene expression changes in β-catenin-knockdown cells were analyzed by qRT-PCR as described (Xu et al., 2010) except that ribosomal 18S RNA was used as a normalization control. The fold change in target genes relative to the control was determined by 2^-∆∆Ct^ method. *p<0.05. (D) Soft agar assays were performed by seeding cells in 0.3% agarose and was quantified at day 14 (i.e. five fields from triplicate wells). Data are means ± SD of three independent experiments. *p < 0.05.

The impact of β-catenin knockdown on several *in vitro* biological properties associated with TNBC tumorigenesis was assessed. To address tumor cell migration, β-catenin shRNA and control TNBC cells were subjected to wound filling assays. Stable knockdown of β-catenin significantly suppressed migration of both MDA-MB-231 and HCC38 cells compared to that of control cells (Fig. 2). The effect was more pronounced in the HCC38 cells. Since the HCC38 has higher level of β-catenin and showed more dramatic phenotypic differences upon β-catenin knockdown, we chose HCC38 cells for further analysis. The stem-like cell population was quantified in β-catenin-knockdown and control HCC38 cells using an ALDEFLUOR assay and flow cytometry. As shown in a representative plot and in the graph of means from two experiments in Fig. 3A and 3B, control HCC38 cells had approximately 30% ALDEFLUOR-positive cells, while β-catenin-knockdown cells had fewer than 5% ALDEFLUOR-positive cells. Consistent with this finding, the expression of β-catenin/TCF target gene, and stemness regulator, *Bmi-1*, was significantly lower in β-catenin-knockdown cells compared to control cells. Other β-catenin/TCF targets, including *Axin2* and *c-Myc*, were similarly downregulated in β-catenin-knockdown cells, although levels of the target *Tert* was not significantly changed (Fig. 3C). Lastly, similar to migration and stemness, colony formation in soft agar was significantly reduced in β-catenin-knockdown HCC38 cells compared to control cells (Fig. 3D). Collectively, these data indicate that β-catenin activity is required for many of the tumorigenic characteristics of TNBC cells, including cell migration, stem-like cell properties and colony formation. Notably, proliferation was unchanged in β-catenin-knockdown cells (S1 Fig.).

**Figure 4:**
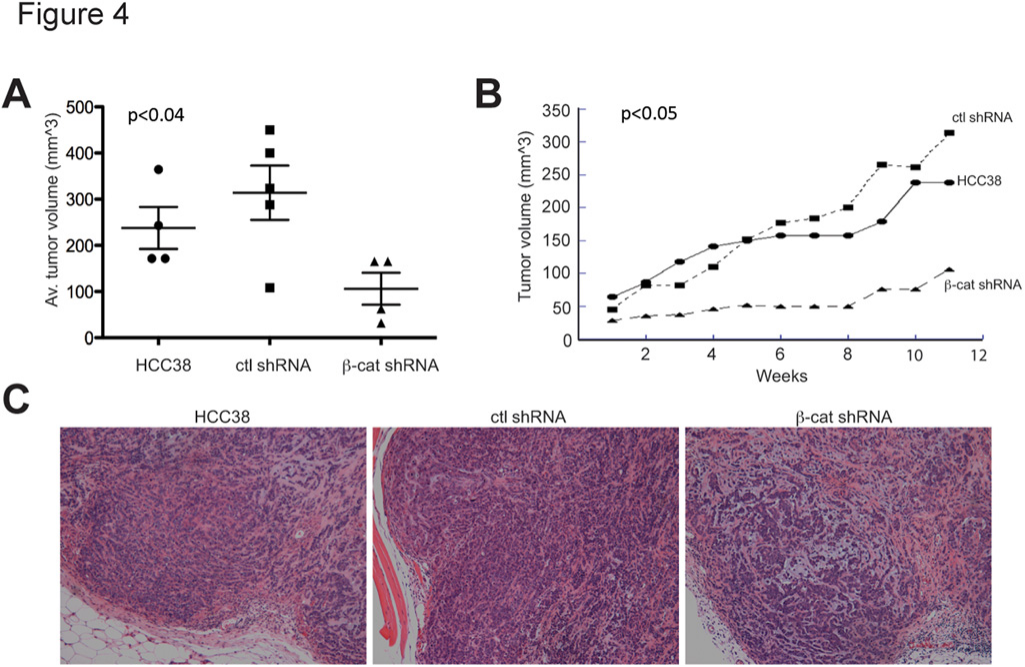
β-catenin knockdown reduces the in vivo tumorigenic potential of HCC38 TNBC cells. (A) 5x10^^6^ parental, scrambled control shRNA or β-catenin-knockdown cells were injected with matrigel into the mammary fat pad of Nu/Nu female mice (n = 10 per group) and were sacrificed 11 weeks later. Dot plot of tumor volume for tumor-bearing mice is shown with mean per group demarcated by the line. p<0.04 between β-catenin-knockdown and control cells by ANOVA. (B) Tumor growth over 11 weeks after implantation and biweekly measurements. p<0.05 between β-catenin-knockdown and control cells by ANOVA. (C) Histopathology of tumors from mice injected with β-catenin-knockdown and control. Tumors were fixed, sectioned and stained with H&E. Magnification, 10X.

We next tested the requirement for β-catenin in the *in vivo* tumorigenic potential of HCC38 TNBC cells upon orthotopic transplantation (n = 10 per group). As shown in Fig. 4A and 4B, the average tumor volume and growth were significantly decreased for cells with β-catenin knockdown compared to parental and control shRNA cells. The tumors that formed in mice injected with β-catenin-knockdown cells were very small and necrotic (Fig. 4C). These data indicate that β-catenin is necessary for TNBC initiation and growth *in vivo.*

**Figure 5:**
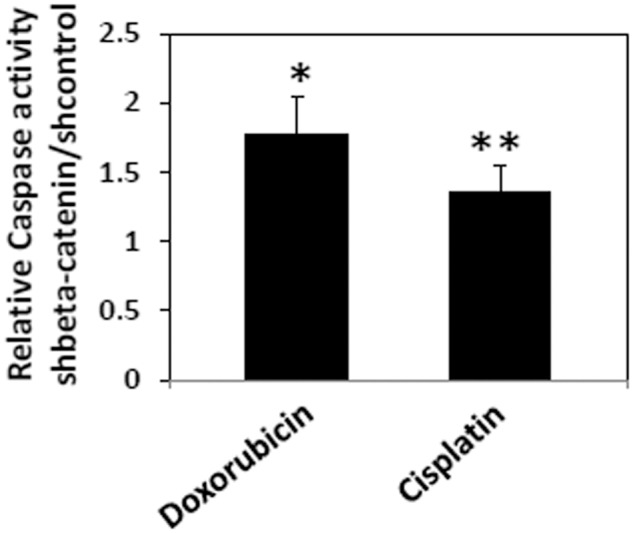
β-catenin contributes to the sensitivity of TNBC cells to chemotherapeutic agents. β-catenin-knockdown or control HCC38 cells were treated with 0.5 μM doxorubicin or 3.0 μM cisplatin for 24hr and apoptosis was measured using the Caspase 3/7 Glo assay. Relative Caspase activity was shown as β-catenin-knockdown over control. Bar represents means ± SD. *p<0.01, **p<0.05.

Finally, the impact of Wnt/β-catenin signaling on the chemotherapeutic response of TNBC cells was investigated. Specifically, the sensitivity of β-catenin-knockdown cells was compared to that of control cells each treated with 0.5 μM doxorubicin or 3.0 μM cisplatin, and we found that the β-catenin-knockdown cells were more sensitive to doxorubicin or cisplatin-induced cell death after 24-hour treatment compared to control shRNA cells (Fig. 5). The effect was more profound with doxorubicin treatment compared to cisplatin treatment. It suggests that β-catenin/TCF-mediated gene regulation is important for the chemosensitivity of TNBC cells.

## Discussion

We have previously shown that β-catenin-dependent Wnt signaling is enriched in TNBCs and is associated with reduced overall survival in breast cancer patients [16]. In the present study, we tested the requirement for β-catenin and Wnt signaling in the tumorigenic potential of human TNBC cell lines. We found that β-catenin is necessary for TNBC cell migration, colony formation and stem cell properties *in vitro* and tumorigenesis in immunocompromised mice. In addition, the responsiveness of TNBC cells to chemotherapeutic agents, such as doxorubicin and cisplatin, is dependent on β-catenin and Wnt pathway status.

It has been reported that β-catenin signaling dosage dictates tissue-specific tumor predisposition in *Apc*-driven cancer. In a mouse model, *Apc1638N*, with reduced β-catenin levels, female mice developed mammary tumors instead of gastrointestinal tumors [21]. We found that the dosage of β-catenin was important in downstream target gene regulation and affected several behaviors associated with tumorigenesis and development of TNBC. In stable β-catenin knock-down TNBC cells, reduction of β-catenin levesl did not affect cell growth; yet, other phenotypes, such as cell migration, were significantly impaired. DiMeo *et al.* reported that Wnt signaling was not required for the proliferation of SUM1315 cells in culture as cells overexpressing the Wnt inhibitors SFRP1, DKK1, or shRNA against LRP6, grew at similar rates *in vitro* compared with SUM1315 control cells [22]. Consistent with our findings, Matsuda *et al*. demonstrated that ectopic SFRP1 expression caused a reduction of motility in MDA-MB-231 cells [23]. Together, these data suggest that the Wnt/β-catenin pathway is crucial for controlling cell motility, a property that is significantly associated with metastasis and poor prognosis of TNBC.

Wnt/β-catenin pathway activation has been implicated in stem cell self-renewal, maintenance, and differentiation [22, 24–26]. Many cancers, including breast cancer, contain populations of cells that display stem-like cell properties [9]. In breast cancer, those populations can be marked as CD44^high^/CD24^low^ [27] and ALDEFLUOR-positive [28]. We and others have reported that nuclear and cytosolic accumulation of β-catenin was correlated with high levels of a CD44^high^/CD24^low^ stem cell population in primary TNBC/basal-like breast cancers [16, 29]. In this study, we demonstrated that β-catenin knockdown caused a significant decrease in the ALDEFLUOR-positive stem cell population in TNBC cell lines. In addition, stemness regulators, such as *Bmi-1* and *c-Myc*, were down-regulated in β-catenin-knockdown cells compared to control cells. The epithelial-mesenchymal transition (EMT) promoter and Wnt target gene *Axin2* [14, 30] was also down-regulated. Furthermore, the tumors that formed in mice injected with β-catenin-knockdown TNBC cells were much smaller compared to parental and control shRNA cells. These data suggest that β-catenin is required for cancer stem cell maintenance and EMT features which are also often associated with tumor relapse and metastasis. Thus, targeting β-catenin may have important implications in TNBC therapy since it may perturb the tumor stem cell population that is more resistant to chemotherapy than the bulk of the tumor.

Because TNBCs lack treatment targets due to its triple-negative nature, chemotherapy is the standard treatment. Doxorubicin has been widely used to treat breast cancer especially TNBC [31, 32]. In a neoadjuvant trial, single-agent cisplatin induced response in a subset of patients with TNBC [33]. We found that the β-catenin-knockdown cells were more sensitive to doxorubicin- or cisplatin-induced cell death. These findings indicate that transcriptional activity of β-catenin is important for the chemosensitivity of TNBC cells, and β-catenin may be an attractive therapeutic target for TNBC. There is considerable interest in developing Wnt/β-catenin pathway inhibitors as anti-cancer therapeutics [34]. Recently, it has been reported that treatment of TNBC-derived cell lines and primary mouse tumor cells with small molecule inhibitor ICG-001 blocks proliferation in TNBC cells through down-regulating expression of HMGA2, a regulator of stem cell self-renewal, proliferation and differentiation [35, 36]. We predict that a combination therapy of chemotherapy drugs and Wnt/β-catenin inhibitors may be a more effective way to treat TNBC than with a single agent. Collectively, our findings have significant implications for TNBC patients treated with chemotherapeutics and the development of combination cancer therapy.

## Supporting Information

S1 Fig Stable β-catenin-knockdown does not affect cell growth. MDA-MB-231 (A) and HCC38 (B) cells were seeded in 96-well plates and cell proliferation was measured with MTS assay for 96 hrs. Bar represents means ± SD.
(TIF)
