# Supplementary figures and images for: β-Catenin Is Required for the Tumorigenic Behavior of Triple-Negative Breast Cancer Cells
Source: PLoS One. 2015 Feb 6;10(2):e0117097. doi: 10.1371/journal.pone.0117097 (PMC4319896; doi:10.1371/journal.pone.0117097)

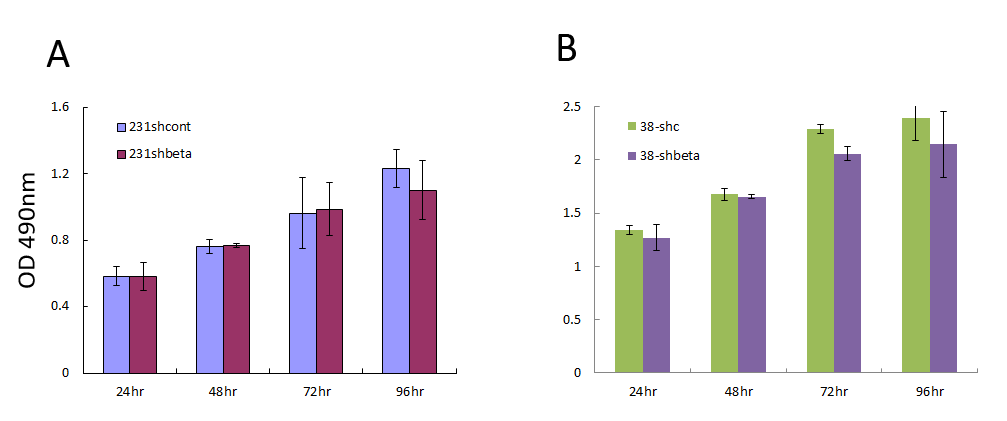

Supplement: S1 Fig — MDA-MB-231 (A) and HCC38 (B) cells were seeded in 96-well plates and cell proliferation was measured with MTS assay for 96 hrs. Bar represents means ± SD. (TIF) [file pone.0117097.s001.tif]
